# Informing climate-smart agriculture in low resource settings for practitioners: A review and analysis of interactive tools

**DOI:** 10.12688/gatesopenres.15299.1

**Published:** 2024-03-01

**Authors:** Daniel Lapidus, Kirsten Franzen, Caleb Milliken, Tyler Ovington, Jenny Frankel-Reed

**Affiliations:** 1 RTI International, Research Triangle Park, North Carolina, USA; 2 Bill & Melinda Gates Foundation, Seattle, Washington, USA

**Keywords:** Climate tools, Climate smart agriculture, Adaptation, Mitigation, Small scale agriculture, Agricultural development

## Abstract

**Background:**

Agricultural producers in developing countries are uniquely vulnerable to the impacts of climate change and have the least ability to adapt. While there is a growing consensus that more financing and resources are needed to address these impacts, information on how to direct funding and support adaptation is dispersed and difficult to find. Agricultural development stakeholders and investors can leverage increasingly available data from a range of online sources to inform their climate smart agriculture investments, but it is not always clear which data tools are easily accessible and which can support different aspects of their programs.

**Methods:**

This analysis aims to inform stakeholders how different tools can inform their climate smart investments. Hundreds of interactive tools were reviewed from multiple sources and a set of criteria was developed to simplify and elucidate the landscape of resources available that support adaptation and GHG mitigation for agricultural producers in low-income countries. The search strategy included a literature review, discussions with key stakeholders, and a review of existing databases of tools (e.g., NDC Partnership Toolbox).

**Results:**

Ultimately 29 tools were identified and compared in terms of how they address both climate risk, adaptation, and mitigation. The data sources behind the tools were also compared, and illustrative user groups were identified. Many valuable, easy-to-use tools exist offering non-climate experts’ opportunities to gain insights into the relationship between climate and small-scale farming systems. However, the tools available are insufficient and should not be relied upon exclusively for informing investments.

**Conclusions:**

This review provides a valuable resource for those looking to inform investments and programming in small-scale agriculture. This set of tools can provide insights that can be leveraged in various ways for a wide range of users, but they also have considerable limitations. This review can help users understand how these tools can be useful and the types of additional context-specific and local information that should be sought.

## Introduction

Small-scale agricultural producers are at ground zero for experiencing the impacts of climate change. Their livelihoods are based on producing crops that are being directly impacted by climate change, which is expected to continue to follow new and increasingly unpredictable patterns in the coming decades. As temperatures rise, precipitation becomes more variable, and extreme weather events become more frequent, small-scale producers in tropical regions in particular are expected to experience the impacts of these stressors most acutely ( Aragón *et al.*, 2021; Morton, 2007). Small-scale producers are considered especially vulnerable as they have generally low incomes and rely predominantly on agriculture for their livelihoods ( Rosenzweig & Hillel, 2008).

In addition to being a uniquely vulnerable sector, agriculture is uniquely suited to concurrently advancing both mitigation and resilience goals ( Cohn *et al.*, 2017). Indeed, the agricultural sector remains the largest economic sector and employer in low-income countries and is often the largest source of emissions ( FAO, 2012). Due to this important role, even though countries are not required specifically report on agriculture in their Nationally Determined Contributions (NDCs), 90% of NDCs mention agriculture and 41% mention food security, and it is a priority sector for climate action ( Schulte *et al.*, 2020). At the United Nations 28th annual Conference of the Parties of the UNFCCC in 2023 (commonly known as COP28), the Declaration on Sustainable Agriculture, Resilient Food Systems, and Climate Action recognizes these multiple roles and was signed by 159 countries.

In light of these needs, the international community has called for ramping up climate financing to help agricultural producers to confront climate change mitigation and adaptation, but so far, the funding is a tiny fraction of climate financial flows and has been deemed wildly insufficient compared to the needs, especially in reaching small-scale producers ( Chiriac *et al.*, 2023; Macquarie *et al.*, 2020). There are growing climate finance opportunities, but project proponents and funders need data and tools to support the design and prioritization process. Policy and planning tasks for NDCs, national, subnational, and sectoral climate action plans and policies also require support of these resources.

The term “climate-smart agriculture (CSA)” has been adopted by the Food and Agriculture Organization (FAO) and other organizations to describe an approach “ *that helps guide actions to transform agri-food systems towards green and climate resilient practices… It aims to tackle three main objectives: sustainably increasing agricultural productivity and incomes; adapting and building resilience to climate change; and reducing and/or removing greenhouse gas emissions, where possible*” ( FAO, 2013; Lipper *et al.*, 2014).

In recent years, there has been a proliferation of data and tools to help address climate-smart agriculture and inform decision-makers on topics at the intersection of climate and production agriculture in low-resource settings. The tools have many intended audiences and purposes, such as resilience or adaptation, mitigation, productivity, sustainability, and more; and many cover combinations of these climate and livelihood related goals. The Nationally Determined Contribution (NDC) Partnership has collected and categorized thousands of tools, guidance documents, platforms, and other resources related to climate action. There are 81 items in the NDC Partnership’s Climate Toolbox labeled as relevant to agriculture ( NDC Partnership, 2023).

Agriculture is a highly site- and context-specific sector. Weather, soil, water, local technology, management practices, and knowledge vary significantly from one farm to the next. This makes the design of tools that are useful for a broad range of agricultural practitioners and investors especially challenging. This variability and complexity limit the capability of tools to inform the implementation of projects that benefit a wide range of producers. Nonetheless, collectively these tools can shed light on the relationship between climate and farming systems. Using the right tools can allow practitioners to screen for climate risks and quickly identify potential solutions and where more analysis may be needed.

Previous research efforts have shed light on existing climate and agriculture tools, as well as screening processes that leverage tools alongside qualitative inputs and steps. Brown (2017) documented and catalogued the climate screening processes and tools that major international agricultural funders implement, including the World Bank, United States Agency for International Development (USAID), UK Department for International Development, African Development Bank, the Swiss Agency for Development and Cooperation, and the Green Climate Fund ( Brown, 2017). Separately, Douxchamps reviewed tools specifically designed for monitoring and evaluating climate resilience in agricultural development, with a specific focus on indicators and methods of measurement ( Douxchamps *et al.*, 2017). Lastly, Neset reviewed map-based tools that support climate change adaptation and are freely accessible on the web; however, this review did not focus on developing country-specific contexts, and it only considered tools related to adaptation to climate change ( Neset *et al.*, 2016).

In this review, the objective is to analyze climate-smart agricultural tools and create a fit-for-purpose categorization that can benefit decision-makers focused on small-scale producers in low-income contexts and help the research community better coordinate their efforts and build on existing tools. The focus is on interactive and accessible tools because these are the most available and convenient for a wide variety of users. Each tool addresses different aspects of climate-smart agriculture as it relates farming in low-resource settings. The purpose of this review is to lay out which tools are relevant for which purposes, enabling users to more quickly access tools that are useful to them. The review also seeks to highlight key limitations of available tools, including their underlying data gaps and use cases where the tools alone are insufficient in answering practitioners’ questions.

## Methods

Given the many available tools available, and the many disparate uses of each tool, clear and targeted inclusion/exclusion criteria, a search strategy, and comparable classifications were needed to guide the analysis.

*a)*  *Inclusion/exclusion criteria for tools:*
  Multiple selection criteria were used to set boundaries for the analysis. Each tool reviewed would need to meet all four selection criteria to be included:
1. **The tool addresses the intersection between climate and the food system.** This includes tools related to climate impacts, adaptation, or mitigation and intersection anywhere along the food value chain (from producer to consumer) or with food market systems. Tools that focus only on the intersection between the food system and non-climate environmental issues – or tools that focus only on climate and non-food systems – are not included. For example, several tools were found to provide in depth analysis and projection of climate impacts but lacked any information on agriculture (e.g., the Intergovernmental Panel on Climate Change (IPCC) Working Group I (WGI) Interactive Atlas tool); tools like this were not included in the results.
2. **The tool is interactive.** This includes any software, web-based explorer, or other digital feature that is designed for external users to interact with it in some way. This does not include screening or process guides, static narratives, or even interactive reports or story maps. It would also not include raw data related to climate or agriculture. This meant excluding guidance documents, frameworks, country profiles, policy documents, and other useful source materials for analyzing and implementing climate smart agriculture.
3. **The tool explicitly targets at least two developing country contexts**. For tools to be included, they must be applicable to small-scale producer contexts. Although there are small-scale producers in every country, small scale producers are generally more prevalent in low and low-middle income countries. As many countries and other developers have undertaken efforts to produce country-specific tools, only tools covering at least two countries, thus serving a broader audience, were included in this review. Several tools that centered around agriculture and climate but focused only on higher income countries (or were too general to apply practically to developing country contexts) were excluded; we used the World Bank income level classification to discern which countries are considered low income or low-middle income ( Hamadeh *et al.*, 2022).
4. **The tool is readily accessible.** Any tool or resource that requires more than a free sign-up are excluded from this analysis. In addition to being free, it must be accessible to a general audience. For example, Winrock’s Agriculture, Forestry and other Land Use (AFOLU) calculator was excluded because it is only available to implementers of USAID projects. Some climate models and tools were excluded because they were deemed to require too much expertise to be useful to a practitioner. Multiple tools were excluded that required familiarity with geospatial or statistical data and software including ArcGIS or the General Algebraic Modeling System (GAMS).
*b)*  *Search strategy:*
  Several methods were employed to identify relevant climate and agricultural tools. Publication databases were searched since 2015 using the following keywords: “Climate; Review OR Systematic Review; Risk(s); Tool OR Tools OR Toolbox; Agriculture OR Food security; Screening OR Screen; Decisions OR decision-making; Mitigation OR adaptation OR vulnerability; Africa OR Asia OR South America OR Latin America OR low-income country(ies)”.
  Other sources included in the initial search were used or identified through consultation with experts working in the climate adaptation and agriculture space. These included any tools mentioned in conversations between authors and development agencies, funders, and international organizations that were aware of relevant tools being used by practitioners. Finally, toolboxes and resource repositories of institutions such as the World Bank, FAO, and the Consultative Group on International Agricultural Research (CGIAR) system were searched for relevant tools. The most extensive toolbox used was created by NDC Partnership and profiled climate sources as they relate to nationally determined contributions. The NDC Partnership Climate Toolbox is a database of platforms, guidance documents, and advisory support to help countries plan their NDCs; all tools in this database that were relevant to agriculture were reviewed ( NDC Partnership Climate Toolbox, 2023).
  Once all sources were compiled, a modified snowball approach was used to search for relevant tools in affiliated publications, organizations, and projects from these initial sources. For instance, organizations such as the World Bank, FAO, CGIAR, and the University of Wageningen were identified as potential sources, and the websites of these organizations were scanned for relevant tools. From scanning literature, research organizations’ toolboxes and platforms, and expert input, over 900 total sources were identified and considered. The NDC toolbox housed several of the tools identified, but many tools were excluded from the results of this effort as they did not meet the inclusion criteria.
  The search that was conducted, though extensive and far reaching, is non-exhaustive. There are simply too many tools that have been developed by too many developers to ensure that all are being captured. Additionally, some subjectivity was used in determining whether a tool met any specific criteria.
*c)*  *Tool characterization criteria:*
  Once the tools were filtered to a shortlist, they were characterized according to multiple criteria that were considered comparable across tools and conducive to analysis. Note that although clear criteria were identified, the characterization was subjective based on the authors’ review of each tool. See Table 1 for detailed information on criteria used for characterization of the tools.

**Table 1.** Characterization criteria for tools.

Characterization criteria for tools	
**Description**	A simple paragraph of 2-3 sentences was used to describe what the tool does and its purpose. These descriptions are in the words of the authors of this review unless otherwise stated.
**Use cases**	Tools were classified according to what primary and secondary questions they might help answer. These questions reflect the concerns of potential users that would be addressed by the tool.
**Intended user**	Five archetypes or groups were identified to shed light on which tools were more appropriate for which kind of users. The five archetypes were “Sustainable sourcing manager”; “Implementing partner activity lead”; “Agricultural development funder”; “Agricultural policy maker”; and “Agricultural researcher”. Each group was listed in order of relevance to the tool.
**Risk Relevance (exposure, ** **hazards, vulnerability, ** **adaptive capacity, adaptive ** **solutions adaptive response)**	Climate risk is a function of exposure, hazards, and vulnerability, and response ( Lavell *et al.*, 2012). Tools that focused on climate risk were reviewed for whether they addressed exposure, hazards, and vulnerability, and *adaptive* responses. Tools were also reviewed according to the degree to which they identified adaptive capacities (e.g., factors that may allow people to adjust or respond to potential risks) and adaptive solutions including actions people could take to mitigate risk).
**Mitigation relevance**	Tools that addressed GHG mitigation were classified according to what aspect of mitigation they were focused on (e.g., emission profiles, mitigation options)
**Crops**	Many tools identify or provide data on specific crops. Where crops were specifically considered by the tool they were identified.
**Scale**	Tools were categorized according to their geographical specificity, ranging from global, national, sub- national, to field level. “Sub-national” is meant to capture any geographical level that is considered larger than the field level but smaller than national level.
**Outputs**	Many tools simply allowed users to explore data on maps, but others provided GHG emission data, land use indicators, or other metrics.
**Ease of use**	Interactive tools generally fit three categories: *simple*, *moderate* and *complex*. Simple typically refers to: “point and click” types of tools where you can turn on and off layers and scroll on a mapping interface. Moderate typically refers to “plug and play” type of tools where you have to enter some information but typically not an extensive amount. A complex categorization was used when the analysis was more of a “detailed, deep dive” that required significant data inputs from the user or some training to use the tool. Tools classified as *simple* are likely to be more easily navigated and digested, while *moderate* tools would take more time depending on how much project level data was available. Generally, a greater investment of time is needed as the user moves from a simple to *complex* tool.

Lastly, a binary yes or no classification was used for whether each tool provided historical data, future projections, data source documentation, methodology documentation, or evidence of recent updates (>2020).

For tools that include climate impacts, data sources used by each tool were reviewed. Often, tools use data produced or sourced by the publishing institution, and many tools used various data sources that were not used by other tools characterized in this review. However, where underlying data was used by more than one tool characterized herein, these common data were identified in the results in order to shed light on the most common sources of land use, socioeconomic, and climate data that are used by these tools.

Based on careful consideration of the tools’ apparent intended users (sometimes explicitly cited on the website), or who the tools could provide valuable information to, five user archetypes were created: Implementing Partner, Agricultural Development Funder, Agricultural Policy Maker, Sustainable Sourcing Manager, and Agricultural Researcher. For further description of each of these user groups, see Table 2. Tools were also characterized by how they relate to climate change based on whether they incorporate climate risk (exposure, hazard, vulnerability) or measure mitigation potential ( Table 3).

**Table 2.** User Group Definitions.

*User Group*	*Definitions*
***Implementing*** *** Partner***	*This user group refers to an organization or individual that partners with a funder or investor in small-scale agriculture* * that leads the on-the-ground implementation of a portfolio/program/project. Example users are non-governmental * *organizations, agribusinesses, agricultural extension service providers, and other receivers of agricultural development * *grant funding. These users could prioritize welfare, livelihood, and gender equity within small holders. They could be* * interested in incorporating best-available climate and agricultural data and tools into their implementation efforts* * – including to inform decisions like where to focus (e.g., in terms of which commodity value chains, in terms of where* * along the value chain, in terms of geographic regions) and how best to enable more resilience across food systems.*
***Agricultural *** ***Development*** *** Funder***	*This user group refers to an organization or individual that invests in agricultural development in low- and lower-* *middle income countries. Some example users are philanthropic donors, development agencies or other organization* * that invests funds agricultural development. They could be interested in understanding areas (e.g., geographic areas) at * *highest-priority for deploying new investment dollars, the highest priority needs within these areas, and the best way to* * ensure more resilient food systems result from their funding.*
***Agricultural *** ***Policy Maker***	*This user group refers to government officials and supporting partners involved in the creation of agricultural* * policies. These users are often working to develop the enabling environment of agricultural production through policy* * interventions.*
***Sustainable *** ***Sourcing Manager***	*This user group refers to users who work to sustainably source agricultural products along the supply chain – including * *those sourcing raw materials and value-added agricultural products. These users prioritize sustainability to meet* * expectations of their consumers and ensure future raw material availability at a desirable price.*
***Agricultural *** ***Researcher***	*This user group captures a wide range of academic or research organization agricultural scientists and researchers* * that would benefit from utilizing the kind of data that is available from climate and agriculture tools. These users could * *focus on understanding areas of vulnerability or exposure for smallholders of select crops or livestock. They could focus* * on the relationships between climate change, agricultural mitigation and adaptation practices, and smallholder welfare * *and gender equity.*

**Table 3.** Key climate terms and examples.

*Key Term*	*Definitions ( IPCC, 2022) and Examples*
***Hazard***	*A hazard is “the potential occurrence of a natural or human-induced physical event or trend that may cause loss * *of life, injury, or other health impacts, as well as damage and loss to property, infrastructure, livelihoods, service* * provision, ecosystems, and environmental resources.” Indicators representing hazards included propensity* * for natural disasters, including storms, flooding, drought, extreme heat, and other climate-related drivers of * *potential destruction or harm.*
***Exposure***	*Exposure is “presence of people; livelihoods; species or ecosystems; environmental functions, services, and* * resources; infrastructure; or economic, social, or cultural assets in places and settings that could be adversely* * affected.” Examples of indicators representing exposure in the tools include area under cropland production, * *yield of specific crops or livestock, or population densities in a geography.*
***Vulnerability*** *** (+ adaptive *** ***capacity)***	*Vulnerability is the “propensity or predisposition to be adversely affected”. Vulnerability encompasses a variety* * of concepts and elements, including sensitivity or susceptibility to harm and lack of capacity to cope and adapt. * *Indicators assessing vulnerability can relate to human capital (e.g., literacy or education rates) or biophysical* * factors (e.g., soil quality). Other indicators that reflect vulnerability are access to goods and services such as * *access to banking, broadband, markets, or health facilities. Indicators that reflect adaptive capacity are also * *included in this category as adaptive capacity is the positive side of vulnerability (e.g., low access to broadband is * *vulnerability; high access is adaptive capacity).*
***Adaptive Response***	*The IPCC has a more expansive definition of “responses” that include biophysical and natural responses of * *climate change; for simplicity this study narrows responses to human adaptive responses, which is meant to* * capture human intervention to facilitate adaptation. Adaptive solutions include specific measures (e.g., using* * climate smart crops or livestock breeds, introducing buffers to prevent soil and water erosion). Some adaptive * *solutions are captured at the national level in tools such as ClimateWatch, others are more specifically linked to * *crop and livestock production system exposure, hazards, and vulnerabilities, as found in CRISP.*

## Results

A total of 29 tools were identified that met the criteria (see Table 4). Ten were from the NDC Toolbox, ten from the snowball approach, and nine from expert consultations. Out of 29 tools, 20 considered at least one aspect of risk (i.e., exposure, hazard, vulnerability), 11 were focused on climate mitigation, and three tools included aspects of both mitigation and risk (the CSA Programming and Indicator Tool, the Climate Risk Toolbox, and ClimateWatch).

**Table 4.** Tool names, websites, and author(s).

Name of Tool	Website	Author(s)
**Agricultural Adaptation (AgriAdapt) Tool**	https://www.agriadapt.org/	WRI
**Climate Impact Viewer**	https://a-plat.nies.go.jp/ap-plat/asia_pacific/index.html	Asia-Pacific Climate Change Adaptation Information Platform
**Climate Analysis Indicators Tool—CAIT ** **2.0 (Climate Watch)**	https://www.wri.org/our-work/project/cait-climate-data-explorer	WRI
**Climate Change, Agriculture and Food ** **Security (CCAFS) Mitigation Option Tool**	https://ccafs.cgiar.org/resources/tools/ccafs-mot-mitigation-options-tool-agriculture	LEDS Global Partnership
**Cool Farm Tool**	https://coolfarmtool.org/	Cool Farm Alliance, Sustainable Food Lab
**Ex-Ante Carbon-balance Tool (EX-ACT) ** **(includes EX-ACT VC)**	https://www.fao.org/in-action/epic/ex-act-tool/en/	FAO
**Land-use Planner**	https://landuseplanner.org/	EU REDD Facility
**EarthMap**	https://earthmap.org/	OpenForis
**Resilience Atlas**	https://www.resilienceatlas.org/	Conservation International
**The Global Livestock Environmental ** **Assessment Model interactive (GLEAM-i)**	https://gleami.apps.fao.org/	FAO, International Finance Corporation, World Bank
**AgMIP Global Gridded Crop Model ** **Intercomparison Project (GGCMI)**	https://agmipimpactsexplorer.wenr.wur.nl/ggcmi-maps	University of Wageningen
**AgMIP IFPRI Impacts viewer**	https://agmipimpactsexplorer.wenr.wur.nl/ifpri-impact-viewer	University of Wageningen
**Climate Change Knowledge Portal**	https://climateknowledgeportal.worldbank.org/	World Bank
**EX-Ante Carbon-balance Tool for value** ** chains (EX-ACT VC)**	https://www.fao.org/in-action/epic/ex-act-tool/suite-of-tools/ex-act-vc/en/	FAO
**Aqueduct (including Aqueduct Food)**	https://www.wri.org/aqueduct	WRI
**Agricultural Adaptation Atlas**	https://adaptationatlas.cgiar.org	CGIAR
**Carbon Benefits Project**	https://cbp.nrel.colostate.edu/	UNEP
**Climate Risk Toolbox**	https://data.apps.fao.org/crtb/	FAO
**CSA Programming and Indicator Tool**	https://ccafs.cgiar.org/resources/tools/csa-programming-and-indicator-tool	CGIAR
**Global Information and Early Warning ** **System on Food and Agriculture**	https://www.fao.org/giews/earthobservation/index.jsp	FAO
**Trase Supply Chains**	https://supplychains.trase.earth/	Stockholm Environment Institute (SEI), Global Canopy
**Global Agricultural & Disaster ** **Assessment System (GADAS)**	https://geo.fas.usda.gov/GADAS/index.html	USDA
**Agro-Chain Greenhouse Gas Emissions** ** (ACE) calculator**	https://cgspace.cgiar.org/handle/10568/106161	CGIAR
**FLW Value Calculator**	https://www.flwprotocol.org/why-measure/food-loss-and-waste-value-calculator/	Quantis
**Climate Impact Explorer**	https://climate-impact-explorer.climateanalytics.org/	Climate Analytics
**CRISP**	https://crisp.cgiar.org/	CGIAR
**Food Systems Dashboard**	https://www.foodsystemsdashboard.org/	The Global Alliance for Improved Nutrition
**RegioCrop**	https://regiocrop.climateanalytics.org/choices	Climate Analytics
**Climate Vulnerability Monitor ** **– Biophysical Data Explorer**	https://climatevulnerabilitymonitor.org/biophysical/	V-20

***Climate risk:*** Within the climate risk category, most of the 20 tools included functionality to assess an agricultural-related exposure (e.g., maize yield) to an explicitly climate-driven hazard (e.g., increased temperature or more variable precipitation). Under this category, map-based “simple” tools were the most common type. This type of tool enables users to explore the relationships between climate-related hazards such as temperature, precipitation, extreme weather events, floods, and water scarcity against agricultural-related exposures such as land use, cropland coverage (often by type of crop), or productivity. Examples of lighter touch, simple tools include Resilience Atlas, the World Bank Climate Change Knowledge Portal, and Agricultural Model Intercomparison and Improvement Project (AgMIP) International Food Policy Research Institute (IFPRI) Impacts Viewer. Of these 20 climate risk tools, 15 also provided functionality to assess vulnerability and adaptive capacity, but only four tools provided any way to assess or compare adaptive solutions. Adaptive capacity and vulnerability data generally took the form of socioeconomic data such as income or poverty levels, food security, education levels, or access to beneficial services, as well as biophysical adaptive capacity, e.g., soil quality. Some example tools are AgriAdapt, Climate Vulnerability Monitor, Resilience Atlas, Food Systems Dashboard, Agriculture Adaptation Atlas, Climate Risk Toolbox, Global Agricultural & Disaster Assessment System, and CSA Programming and Indicator Tool.

Tools addressing adaptive solutions generally are more qualitative and help users understand the relationships between environment, activity, and potential outcomes. The five tools that offered users the ability to explore adaptive solutions were the Agriculture Adaptation Atlas, CSA Programming and Indicator Tool, ClimateWatch, the “Climate Risk Planning & Managing Tools for Development Programmes in Agrifood Systems” (CRISP), and RegioCrop ( Table 5). These are relatively new tools that have been designed precisely to offer solutions; they also allow users to download relevant data for more detailed or bespoke analyses.

**Table 5.** Tool Indicators.

Name of Tool	Exposure	Hazards	Vulnerability (+ adaptive capacity)	Mitigation	Field-level mitigation	Adaptative solutions	Ease of Use		
							Simple	Moderate	Complex
**Agricultural Adaptation (AgriAdapt) Tool**	✓	✓	✓				✓		
**Climate Impact Viewer**	✓	✓					✓		
**Climate Analysis Indicators Tool—CAIT 2.0 (Climate Watch)**	✓		✓	✓		✓	✓		
**Climate Change, Agriculture and Food Security (CCAFS) Mitigation Option** ** Tool**				✓	✓			✓	
**Cool Farm Tool**				✓	✓				✓
**Ex-Ante Carbon-balance Tool (EX-ACT) (includes EX-ACT VC)**				✓	✓				✓
**Land-use Planner**				✓	✓				✓
**EarthMap**	✓	✓					✓		
**Resilience Atlas**	✓	✓	✓				✓		
**The Global Livestock Environmental Assessment Model interactive (GLEAM-i)**				✓	✓			✓	
**AgMIP Global Gridded Crop Model Intercomparison Project (GGCMI)**	✓	✓					✓		
**AgMIP IFPRI Impacts viewer**	✓		✓				✓		
**Climate Change Knowledge Portal**	✓	✓	✓				✓		
**EX-Ante Carbon-balance Tool for value chains (EX-ACT VC)**				✓	✓			✓	
**Aqueduct (including Aqueduct Food)**	✓	✓	✓				✓		
**Agricultural Adaptation Atlas**	✓	✓	✓			✓	✓		
**Carbon Benefits Project**				✓	✓			✓	✓
**Climate Risk Toolbox**	✓	✓	✓				✓	✓	
**CSA Programming and Indicator Tool**	✓	✓	✓	✓		✓		✓	
**Global Information and Early Warning System on Food and Agriculture**	✓	✓					✓		
**Trase Supply Chains**	✓						✓		
**Global Agricultural & Disaster Assessment System (GADAS)**	✓	✓	✓				✓	✓	
**Agro-Chain Greenhouse Gas Emissions (ACE) calculator**				✓				✓	
**FLW Value Calculator**				✓	✓			✓	
**Climate impact explorer**	✓	✓	✓				✓		
**CRISP**	✓	✓	✓			✓	✓		
**Food Systems Dashboard**	✓	✓	✓				✓		
**RegioCrop**	✓		✓			✓	✓		
**Climate Vulnerability Monitor**	✓	✓	✓				✓		
**TOTAL**	20	16	15	11	8	5	19	9	4

***Climate mitigation:*** For the 11 tools focused on climate mitigation, most (eight) allowed users to examine the GHG impacts of field-level interventions ( Table 5). Other tools had a variety of different foci. For example, one tool each examined emissions at the national level (ClimateWatch); in a commodity supply chain; from a particular food product (ACE Calculator); and related to food loss and waste (FLW Value Calculator). These tools generally require more input data for analysis than simple tools but produce quantifiable impacts on GHG emissions and sometimes also yields, food security metrics, or biodiversity outcomes. For these tools to be informative, they typically require more detailed input data and knowledge of specific geographies, farming practices, and/or business management practices.

No tools attempted to address both risks and mitigation in a robust, holistic, or integrated way. The only tool that addressed both mitigation and adaptation was a CSA programming and indicator toolbox, intended to support monitoring, learning, and evaluation types of activities rather than to support agricultural decision-making directly.

***Private sector supply chains and/or value chains:*** A topic that cut across climate risk and climate mitigation is related to tools designed to address commercial agricultural supply chains for specific crops or livestock products. Most of the tools categorized as climate risk also have the capability of providing insights on the vulnerability of crops in commercial supply chains. Two tools that explicitly address this functionality and provide user stories to showcase this usage are World Resources Institute (WRI)’s Aqueduct and AgriAdapt tools. The former has numerous examples of how companies have used the tool to help them better understand how climate-related water risks may play into sourcing decisions ( WRI Aqueduct User Stories, 2023). The latter is calibrated to understand risks to specific supply chains, including Colombian coffee, Indian paddy rice, and Indian cotton.

There are six mitigation focused tools that explicitly address how private sector actors can incorporate GHG emission tradeoffs into their planning. These include the Cool Farm Tool, EX-Ante Carbon-balance Tool for Value Chains (EX-ACT VC), Agro-Chain Greenhouse Gas Emissions (ACE) calculator, Food Loss and Waste (FLW) Value Calculator, Trase Supply Chains, and the Global Livestock Environmental Assessment Model (GLEAM). They each have different commodity and value chain orientation and relevance, but each share the functionality of being able to estimate GHG emissions along a value chain as long as the user provides the right inputs. More detailed tools might require input data such as livestock head, feed type composition, soil or water management practices, fertilizer use, days of cultivation of crops, area of land use or land use change, etc.

**Commonly used datasets to spatially explore risk:** Tools that use data from externally produced data products rely on a limited number of sources for climate, crop, and socioeconomic data. Any data sources that were referenced in more than one risk tool are highlighted in Table 6. Most of these underlying datasets are trusted sources of widely available data used to spatially depict or model key metrics or variables representing exposure, hazard, or vulnerability. Tools that included external sources primarily used agricultural production data from FAO or IFPRI, socioeconomic data from the World Bank or USAID, and climate and environment data from the National Aeronautics and Space Administration (NASA), the National Oceanic and Atmospheric Administration (NOAA), CLIMate ADAptation (CLIMADA), and the Inter-Sectoral Impact Model Intercomparison Project (ISMIP).

**Table 6.** Datasets used by multiple tools in the analysis.

Data source	Data producer	Metrics/variables	Tools citing	Data available
**CLIMADA**	ETH Zurich	Natural disasters, wildfires, flooding, precipitation, temperature, exposure to crop failure	AgriAdapt, Climate Impact Explorer	Variable (dependent on data layer)
**Inter-Sectoral Impact** ** Model Intercomparison ** **Project (ISMIP)**	Potsdam Institute for Climate Research	Natural disasters, wildfires flooding, heat waves, heat stress, exposure to crop failure	AgriAdapt, Climate Impact Explorer, RegioCrop	Variable (dependent on data layer)
**Climate Hazards Group ** **InfraRed Precipitation** ** with Station data (CHIRPS)**	University of California, Santa Barbara	Precipitation, temperature	Global Agricultural and Disaster Assessment System (GADAS), Resilience Atlas	Weekly data from 1981 to near- present
**Global Agro-Ecological ** **Zones (GAEZ)**	FAO	Crop suitability by climate zone	CSA indicator tool, AgriAdapt	2010 baseline
**FAOSTAT**	FAO	Crop production, crop value, crop area	CSA indicator tool, Resilience Atlas	Annual
**Spatial Production ** **Allocation Model ** **(MapSPAM)**	IFPRI	Crop production, crop value, crop area	Aqueduct, GADAS, Resilience Atlas	Two time steps: 2010, 2017
**Demographic and Health ** **Surveys Program**	USAID	Gender equity, banking access, education, literacy, malnutrition, public health	GFAS, Resilience Atlas, Agricultural Adaptation Atlas	Variable by country

**User groups:** Illustrative user groups were developed to shed light on the kinds of archetypical users that may want to use select tools and how they may derive use of each tool. Five user groups and illustrative examples for how they might use tools to answer different types of questions are described in Table 7.

**Table 7.** How different user groups could utilize the tools.

User Group	Questions They Might Have	Example of How Tool Can Support Answering
**Implementing ** **Partner**	How is the production of cereals projected to change in the next 10 –30 years due to climate? What adaptive solutions exist that could be considered for implementation?	The **Agriculture Adaptation Atlas** allows users to explore the level of climate risk at the national and sub-national level to several different categories of crop types and provides information on 8 different types of adaptive capacities and 19 different options for adaptive solutions for future climate scenarios.
	We’re working to implement a project focused on multiple value chains in Ethiopia. What percentage of the population might be exposed to river floods? What about crop failures?	The **Climate Impacts Explorer** allows users to look sub-nationally at a wide range of acute and chronic physical risks and assess how those might impact different segments of the population for four crops (i.e., maize, rice, soybean, and wheat) at the national and sub-national level based on user-provided data.
**Agricultural ** **Development ** **Funder**	We’re considering different options for climate-smart rice in Indonesia. What are the tradeoffs between different options for reducing GHG emission and emission intensity?	**Climate Change, Agriculture and Food Security (CCAFS)-** **Mitigation Options Tool** can provide a field-level ranking of the most effective mitigation options for over 30 crops according to their mitigation potential in relation to existing management practices and climate and soil characteristics.
	We’re investing in a genetic innovation that should increase water use efficiency, once we prove it’s possible, should we pilot in wheat or cassava? Could climate data help us scope crop selection for a pilot?	**Aqueduct** could help the funder see at the national level where projected water risks exist for nearly a dozen staple crop categories to understand where there may be the greatest upside for water use efficiency improvements in cassava compared to wheat.
**Agricultural Policy** ** Maker**	What are the national drivers of emissions and relevant adaptation and mitigation plans in Uganda?	**Climate Watch** could supply data on national drivers of emissions and key commitments in each sector that the country has made towards the Paris Accord to reduce emissions by 2050.
	What are the main impacts of climate on agriculture and livelihoods in a given country?	The **World Bank’s Climate Change Knowledge Portal** includes narratives and analyses on current climate, projections (across user-tunable emissions scenarios), and extreme events. It also allows the user to download relevant national-level data.
**Sustainable ** **Sourcing Manager**	I work for a coffee company and I’d like to visualize and explore how coffee suitability might change in different regions of the world due to climate change?	**AgriAdapt** allows a user to view the coffee suitability index under different scenarios for an exact location. Also, it could inform a user where warmer and wetter conditions may become more prevalent (and thus potential exposure to pests and diseases) by applying temperature and rainfall layers that show projected change for a certain timeframe.
	What is the deforestation exposure in regions of Indonesia’s palm oil production?	**Trase Supply Chains** can be used to show certain palm oil supply chain actors and/or regions within a country and their level of exposure to deforestation driven by palm oil production.
**Agricultural ** **Researcher**	I am conducting research on how floods might impact the livelihoods of small-scale producers in the coming decades. What datasets should I use?	There are spatial flood datasets referenced in **GADAS**, **Climate ** **Impact Explorer**, and **Climate Risk Toolbox**, and there are livelihood datasets referenced in the **Resilience Atlas**. Downloading these and combining them in ArcGIS with other recent, local data sets can enable research into impacts.
	I’d like to use a conceptual framework to analyze the adaptation options that are relevant to certain combination of hazards, sectors, geographies, and socioeconomic contexts.	The **CRISP tool** has mapped out thousands of combinations of contexts and impact chains to show the many ways in which climate may impact sectors and which adaptive solutions may be relevant to those contexts.

## Takeaways/discussion

***Many valuable, easy-to-use tools exist*** offering non-climate experts and generalists' opportunities to gain interesting insights into the relationship between climate and small-scale producers and farming systems. Tools allow users to screen for climate risks and quickly identify where more digging or research is needed. These tools provide efficiency benefits, allowing users to streamline tasks and freeing up resources to dig deeper or ask other related questions. The tools also enable generalists to better understand concepts related to climate-smart agriculture, and in particular risk and climate mitigation. Integrated, appropriate use of these tools in development efforts (where relevant) can efficiently improve climate outcomes of projects.

***No tool should be used as a standalone resource to comprehensively address climate-smart agriculture as it relates to smallholders***. Tools that are meant to be broadly applicable are often limited in context-specific detail and nuance. They can be helpful for enabling users to broadly screen for potential climate-related impacts, assess alternative approaches for climate-relevant goals, and inform sustainability in agricultural supply chains. However, to fully address any program or intervention, a wide range of experts, stakeholders, resources, and customized analyses are needed alongside tools.

***Researchers may be able to mine the tools to identify and download relevant datasets to conduct more tailored or bespoke analysis.*** One of the most important benefits of these tools is that they can point to the right datasets to be used for further exploration. The developers of these tools have spent significant time curating datasets relevant to climate, agriculture, and smallholder farming systems. However, these tools had to make tradeoffs between data coverage and specificity. Some of the tools reviewed here reflect data that is not the most spatially disaggregated (but may be the best available covering a wide geographical area of focus for the tool) or recent; for example, the Aqueduct tool includes MapSPAM crop data from 2010, which is 13 years old at the time of this publication. To maximally benefit from these tools when conducting bespoke analysis, researchers should consider both what data they can mine from these tools and what additional, more spatially and temporally disaggregated datasets (which often exist from individual projects, statistical agency data, or survey data collection) they can combine with the datasets in these tools to jumpstart efforts to produce tailored or bespoke analysis. Researchers should remember that they should not assume the tools’ data is up-to-date or the best-available data source for a given research question.

***Tools do not collectively address resilience and adaptation planning very well.*** There are many tools that do address adaptation planning and resilience, but they tend to be more process oriented (e.g., a series of questions that guide the user through a process). Since adaptation options are highly specific to context—involving multiple factors such as likelihood or severity of hazards, propensity to be negatively impacted (vulnerability), the presence of valued assets (exposure), and details about the social/political/cultural context—they are necessarily less conducive to a stylized model or interactive tool. Nonetheless, the Agricultural Adaptation Atlas (AAA) and CRISP do characterize and display different types of adaptation options, and the AAA and Resilience Atlas incorporate relevant adaptive capacities that may be applicable in modulating climate impacts. The Climate Risk Toolbox includes geospatial data to analyze a target geography’s adaptive capacity. But few other interactive tools attempt to tackle resilience and adaptation in a very actionable way.

***Tools could do a better job of estimating the impact of climate on a wide range of specific crops and at a high spatial resolution.*** Most climate impact tools only model the projected impact of climate on the world’s commonly grown, highly traded staple crops: like wheat, rice, cotton, and soy (e.g., the AgMIP IFPRI Impacts Viewer). Some global staple crops, such as wheat, rice, soy, and cotton, have spatial data at higher resolutions of 30 m × 30 m or general cropland at 10 m × 10 m. Models that use IFPRI’s MapSPAM dataset, such as the Resilience Atlas, GADAS, and Aqueduct, allow the user to explore 42 crops at coarser spatial resolution of 10 km × 10 km. Although the data and methods for estimating the productivity impacts of climate on a wide range of crops is now available and presumably a tool could be built to screen how these crops may become more, or less, impacted or suitable with climate change, such a tool was not identified. Tools estimating GHG emissions tend to have the capacity to be more crop explicit, depending on geography and data available for a specific investment.

***Climate-smart agricultural tools generally do not cover a wide range of environmental impacts.*** Tools tend to focus on the impact of climate on productivity, or the impact of agricultural interventions on GHG emission. Fewer go further to assess how agricultural interventions or climate may impact other environmental outcomes such as biodiversity, soil, and water quality. Cool Farm Tool and the EU Reducing Emissions from Deforestation and Forest Degradation (REDD) Facility’s Land-use Planner include some information regarding biodiversity and environmental impacts. The Aqueduct tool focuses on assessing the impact of agriculture on water related outcomes. Tool outputs, which include biodiversity or environment-specific impacts, tend to require more detailed input data on farm management and geographic information.

## Conclusion

There is a plethora of interactive tools that share valuable data relevant to climate change and small-scale farming in low-income countries. Here, a non-exhaustive but thorough search identified 29 interactive and accessible tools out of hundreds that were considered. These tools compile data differently to provide valuable insights and details that can be leveraged in various ways to provide needed information to a wide range of users. The most valuable tools are the ones that not only allow information to be displayed but also provide *actionable* insights and clear use cases that allow any user to easily access and understand how the tools can inform their actions. While valuable, it is important to remember that these tools are just that - tools. They should always be used in conjunction with other sources of data and information (e.g., additional data sets, expert consultations, literatures reviews, other targeted research) to inform decision-making.

## Data Availability

All data underlying the results are available as part of the article and no additional source data are required.

## References

[ref-2] Aragón FM Oteiza F Rud JP : Climate Change and Agriculture: Subsistence Farmers’ Response to Extreme Heat. * Am Econ J: Econ Policy.* 2021;13(1):1–35. 10.1257/pol.20190316

[ref-3] Brown DR : Review of climate screening approaches and tools for agricultural investment: Areas for action and opportunities to add value. *CCAFS Working Paper.* 2017. Reference Source

[ref-4] Chiriac D Vishnumolakala H Rosane P : The Climate Finance Gap for Small-Scale Agrifood Systems. *Climate Policy Initiative.* 2023. Reference Source

[ref-5] Cohn AS Newton P Gil JDB : Smallholder Agriculture and Climate Change. *Annu Rev Environ Resour.* 2017;42(1):347–375. 10.1146/annurev-environ-102016-060946

[ref-6] Douxchamps S Debevec L Giordano M : Monitoring and evaluation of climate resilience for agricultural development - A review of currently available tools. *World Dev Perspect.* 2017;5:10–23. 10.1016/j.wdp.2017.02.001

[ref-7] Food and Agriculture Organization: Macroeconomy. In: FAO (ed.), *FAO Statistical Yearbook 2012. * 2012;32–80. Reference Source

[ref-8] Food and Agriculture Organization: CSA Sourcebook: Climate-Smart Agriculture Sourcebook. Executive Summary,2013. Reference Source

[ref-9] Hamadeh N van Rompaey C Metreau E : New World Bank country classifications by income level: 2022-2023. World Bank Data Blog.2022. Reference Source

[ref-10] Intergovernmental Panel on Climate Change: Annex II: Glossary. [Möller, V R. van Diemen, J.B.R. Matthews, C. Méndez, S. Semenov, J.S. Fuglestvedt, A. Reisinger (eds.)]. In: *Climate Change 2022: Impacts, Adaptation and Vulnerability. Contribution of Working Group II to the Sixth Assessment Report of the Intergovernmental Panel on Climate Change.*[H.-O. Pörtner, D.C. Roberts, M. Tignor, E.S. Poloczanska, K. Mintenbeck, A. Alegría, M. Craig, S. Langsdorf, S. Löschke, V. Möller, A. Okem, B. Rama (eds.)]. Cambridge University Press, Cambridge, UK and New York, NY USA,2022;2897–2930. Reference Source

[ref-11] Lavell A Oppenheimer M Diop C : 2012: Climate change: new dimensions in disaster risk, exposure, vulnerability, and resilience. In: *Managing the Risks of Extreme Events and Disasters to Advance Climate Change Adaptation.*[Field, C.B., Barros, V., Stocker, T.F., Qin, D., Dokken, D.J., Ebi, K.L., Mastrandrea, M.D., Mach, K.J., Plattner, G.K., Allen, S.K., Tignor, M., & Midgley, P.M. (eds.)]. A Special Report of Working Groups I and II of the Intergovernmental Panel on Climate Change (IPCC). Cambridge University Press, Cambridge, UK, and New York, NY USA,2012;25–64. Reference Source

[ref-12] Lipper L Thornton P Campbell B : Climate-smart agriculture for food security. *Nature Clim Change.* 2014;4:1068–1072. 10.1038/nclimate2437

[ref-14] Macquarie R Naran B Rosane P : Updated view on the global landscape of climate finance 2019. *Climate Policy Initiative.* London,2020;37. Reference Source

[ref-13] Morton JF : The impact of climate change on smallholder and subsistence agriculture. *Proc Natl Acad Sci U S A.* 2007;104(50):19680–19685. 10.1073/pnas.0701855104 18077400 PMC2148357

[ref-15] NDC Partnership: Climate Toolbox page. Accessed 10/4/2023. Reference Source

[ref-16] Neset T Opach T Lion P : Map-Based Web Tools Supporting Climate Change Adaptation. *Prof Geogr.* 2016;68:103–114. 10.1080/00330124.2015.1033670

[ref-17] Rosenzweig C Hillel D : Climate Variability and the Global Harvest: Impacts of El Niño and Other Oscillations on Agro-Ecosystems. Oxford University Press,2008. 10.1093/oso/9780195137637.001.0001

[ref-18] Schulte I Bakhtary H Siantidis S : Enhancing NDCs for Food Systems: Recommendations for Decision-makers. WWF Germany, Berlin,2020. Reference Source

[ref-19] WRI Aqueduct: User Stories page. Accessed November 29, 2023. Reference Source

