# Supplementary material for: Informing climate-smart agriculture in low resource settings for practitioners: A review and analysis of interactive tools
Source: Gates Open Res. 2024 Mar 1;8:16. [Version 1] doi: 10.12688/gatesopenres.15299.1 (PMC11802523; doi:10.12688/gatesopenres.15299.1)
Supplement: Peer review history

# Reviewer report: Reviewer response for version 1

**DOI:** 10.21956/gatesopenres.16647.r38324

**Published:** 2025-01-07

**Authors:** Laura Cramer

**Affiliations:** 1 International Livestock Research Institute (ILRI), Nairobi, Kenya

This manuscript describes a review of 29 interactive tools related to climate change and smallscale agriculture. The method is described adequately and the tools are analyzed for their potential use by different audiences. The article is well structured and clearly written.

 Some of the citations in the introduction could be updated to more recent publications, especially in paragraph 7.

 The conclusion could be enhanced by adding a recommendation on development of future tools based on the gaps identified in the final three points of the discussion section.

Is the work clearly and accurately presented and does it cite the current literature?

Partly

If applicable, is the statistical analysis and its interpretation appropriate?

Not applicable

Are all the source data underlying the results available to ensure full reproducibility?

No source data required

Is the study design appropriate and is the work technically sound?

Yes

Are the conclusions drawn adequately supported by the results?

Yes

Are sufficient details of methods and analysis provided to allow replication by others?

Yes

Reviewer Expertise:

agriculture development, climate change adaptation and mitigation, linking science with policy

I confirm that I have read this submission and believe that I have an appropriate level of expertise to confirm that it is of an acceptable scientific standard.

## References

[1] : Decision support tools for agricultural adaptation in Africa. *Nature Food* .2024;5(3) : 10.1038/s43016-024-00936-9 186-188 10.1038/s43016-024-00936-9 38443488

# Reviewer report: Reviewer response for version 1

**DOI:** 10.21956/gatesopenres.16647.r38323

**Published:** 2024-10-31

**Authors:** Tamás Mizik

**Affiliations:** 1 Corvinus University of Budapest, Budapest, Hungary

The topic is interesting and of great importance. Climate-smart agriculture is one of the most relevant ways in which agricultural activities can be carried out, especially with limited resources. I have a couple of suggestions and comments:

1. Abstract is too general in its current form. The abstract should state briefly the purpose of the research, the principal results, and major conclusions. An abstract is often presented separately from the article, so it must be able to stand alone.
2. When an abbreviation is explained, no need to repeat it later (Nationally Determined Contribution (NDC)).
3. It would be useful to read more about the creation of user archetypes. How were they identified?
4. The conclusion part is also a bit too general. I would like to read more about the practical impacts of this work.
5. Although a review of the literature is part of the methods applied, only 8 scientific articles were used. Their number needs to be significantly improved.

Is the work clearly and accurately presented and does it cite the current literature?

Partly

If applicable, is the statistical analysis and its interpretation appropriate?

Not applicable

Are all the source data underlying the results available to ensure full reproducibility?

Yes

Is the study design appropriate and is the work technically sound?

Yes

Are the conclusions drawn adequately supported by the results?

Yes

Are sufficient details of methods and analysis provided to allow replication by others?

Yes

Reviewer Expertise:

International agri-food trade, precision and climate-smart agriculture, alternative fuels.

I confirm that I have read this submission and believe that I have an appropriate level of expertise to confirm that it is of an acceptable scientific standard, however I have significant reservations, as outlined above.

# Reviewer report: Reviewer response for version 1

**DOI:** 10.21956/gatesopenres.16647.r37067

**Published:** 2024-07-27

**Authors:** Turyasingura Benson

**Affiliations:** 1 Department of Environment and Natural Resources, Kabale University, Kabale, Western Region, Uganda

Accept this paper for indexing. However, I have the following comments that can improve it.

 1. Authors should show objectives in the abstract and the unconventionality  from the study.

 2. Conclusions should show knowledge generation,

 3. In the INTRODUCTION, Paragraph one needs citations,

 4. All tables should be in APA,

 5. Show the relevance of the study to the SDGs.

 6. All citrated work should be in the reference list. Check kindly.

 7. Add more around 9 current published work from this publisher.

Is the work clearly and accurately presented and does it cite the current literature?

Yes

If applicable, is the statistical analysis and its interpretation appropriate?

Partly

Are all the source data underlying the results available to ensure full reproducibility?

Yes

Is the study design appropriate and is the work technically sound?

Yes

Are the conclusions drawn adequately supported by the results?

No

Are sufficient details of methods and analysis provided to allow replication by others?

Yes

Reviewer Expertise:

Climate-smart Agriculture, climate geomatics, Soil respiration, forest carbon stock, water quality, bibliometrics analysis, R - programing language, crop modeling.

I confirm that I have read this submission and believe that I have an appropriate level of expertise to confirm that it is of an acceptable scientific standard, however I have significant reservations, as outlined above.
